# Giant Cell Arteritis Presenting with Mania, Psychosis, and Cognitive Dysfunction: A Case Report

**DOI:** 10.1155/2023/7989712

**Published:** 2023-02-11

**Authors:** Heidi Madeleine Latvala, Solveig Klæbo Reitan, Arne Einar Vaaler

**Affiliations:** ^1^Department of Addiction, Division of Mental Health and Addiction, University Hospital of Northern Norway, Åsgårdvegen 40, 9016 Tromsø, Norway; ^2^Department of Internal Medicine, Finnmark Hospital Trust, Sykehusveien 35, 9601 Hammerfest, Norway; ^3^Department of Mental Health, St Olav University Hospital, Postboks 3250 Torgarden, 7006 Trondheim, Norway; ^4^Department of Mental Health, Faculty of Medicine and Health Science, Norwegian University of Science and Technology, Høgskoleringen 1, 7491 Trondheim, Norway; ^5^Department of Acute Psychiatry, St. Olav University Hospital, St. Olavs hospital HF, avd Østmarka, Postboks 3250 Torgarden, 7006 Trondheim, Norway

## Abstract

**Background:**

Giant cell arteritis (GCA) is an autoimmune vasculitis affecting medium- and large-sized arteries. Vascular inflammation may lead to narrowing of the arterial lumen, and acute occlusion may result in vision loss and stroke. The classical symptoms include headache, fever, and jaw claudication. However, there is an increasing recognition of atypical presentations. *Case Presentation*. We report a case of a 70-year-old woman presenting with fluctuating manic symptoms and confusion, in addition to headache and musculoskeletal pain. After diagnosis of GCA, treatment with corticosteroids gradually improved the somatic symptoms.

**Conclusion:**

Corticosteroids led to a temporary exacerbation of manic symptoms, which improved after 3 to 4 weeks of continuous treatment, indicating that the symptoms were most likely associated with GCA. The patient manifested with clinical features and a clinical course that has, to our knowledge, not been described or published before. Therefore, GCA may be an underdiagnosed disease in psychiatric populations and should be considered in case of atypical, new-onset psychiatric disorders in the elderly.

## 1. Background

Giant cell arteritis (GCA) is an autoimmune disorder affecting medium- and large-sized arteries. This condition is characterized by granulomas and fused giant cells on histopathology [1]. Additionally, Fc-binding immunoglobulin G complexes have also been described [2]. Although the aetiology has not yet been established, the presence of giant cells indicates an immune reaction to a persistent stimulus, while an autoimmune response to elastin fibres has been suggested as well [2].

Inflammatory granulomas in vessel walls lead to thickening of the intima, which, in turn, leads to narrowing of the lumen, followed by a possible rapid and acute occlusion of the arteries, ultimately leading to the ischaemic damage of various organs [3]. Cranial ischaemic complications include blindness and posterior circulation stroke. Therefore, early recognition of classical as well as nonclassical symptoms is vital. The most common symptoms of GCA are jaw claudication, fever, and temporal headache, as presented in Table 1. More than 40% of patients may have occult manifestations which possibly delay the diagnosis [4]. Typically, GCA symptoms appear suddenly and primarily affect adults older than 50 years of age, and the incidence of GCA has been observed to peak among patients 70 years and older [5]. In Northern Europe, the incidence of GCA is higher (20/100,000 per year for those older than 50 years) than that in Southern Europe (10/100,000 per year for those older than 50 years), and women account for up to 75% of all GCA patients [6].

## 2. Case Presentation

A 70-year-old woman was urgently admitted to the Department of Acute Psychiatry with a one-week history of fluctuating, polymorphic, and predominant symptoms of affective disorders, including irritability, elevated mood, motor agitation, pressured speech, and lack of insight and concern.

Four to three weeks before presentation, the patient experienced persistent insomnia, anorexia, and constipation, three to two weeks before presentation, she was increasingly stiff, and her gait was unsteady. Seven days before hospitalization, the patient had unintentionally lost 5 kg of weight. On the day of hospitalization to the Department of Acute Psychiatry, her clinical condition worsened. The patient appeared confused with disorientation, limited working memory, and disorganised and pressured speech.

The patient's psychiatric history included one episode of delusion in her third decade of life and two episodes of depression in her fifth decade of life. She was treated with psychotherapy and mirtazapine, which led to the gradual remission of her depressive symptoms. She had no history of mania-like behaviour; however, she had a family history of bipolar disorder and Alzheimer's disease. She did not have any relevant history of somatic illnesses.

### 2.1. Investigation

Upon admission to the Department of Acute Psychiatry, an organic cause for her mania was immediately suspected due to the clinical presentation of rapidly developing, polymorphous, and fluctuating psychiatric and cognitive symptoms. Therefore, she was transferred to the Department of Internal Medicine.

The findings upon examination at the time of admission were as follows: temperature 36.9°C, heart rate 103 beats per minute, blood pressure 130/80 mmHg bilaterally, respiratory rate 24 cycles per minute, and an oxygen saturation of 96% on room air. Cardiological, pulmonary, abdominal, neurological, and lower limb examinations were unremarkable. The initial blood test showed increased inflammatory markers and elevated liver parameters, as shown in Table 2.

The chest X-ray showed a possible densification in the left lung, described as a probable postpneumonic finding. However, she reported no recent symptoms of a respiratory tract infection. The investigations revealed that the patient was in a stable somatic condition, and she was readmitted to the Department of Acute Psychiatry, where she was treated with a combination of mood-stabilizers, benzodiazepines, and hypnotics. Her psychiatric symptoms gradually improved over a few days, making her transfer to the general ward possible. Four days after readmission, she developed fever. Since the on-site urine stick test results were positive for nitrates, she was treated with antibiotics (pivmecillinam) for a presumed urinary tract infection, though she did not report any urinary tract infection symptoms. After the initiation of antibiotics, C-reactive protein (CRP) fell from 93 mg/L to 66 mg/L (normal range 0–5 mg/L).

In the general ward, the inflammatory markers were stably elevated; CRP remained at 50 mg/L and ferritin at 420 *μ*g/L. A few liver parameters were also elevated; alkaline phosphatase (ALP) at 120 U/L and gamma-glutamyl transferase (GT) 80 U/L. Electroencephalography (EEG) did not reveal any pathology. Due to reports of back pain and persistently increased inflammatory markers, magnetic resonance imaging (MRI) of the vertebral column was performed, but the findings were unremarkable. The patient also suffered from stiffness, for which polymyalgia rheumatica was suspected, and an erythrocyte sedimentation rate (ESR) was performed. ESR was increased at 83 mm/h (normal range < 25 mm/h for women older than 50 years). The high ESR in combination with increased infection parameters and liver enzymes raised the clinical suspicion of a malignancy, infection, or rheumatic disorder. Antinuclear antibody (ANA) tests, antineutrophil cytoplasmic antibodies (ANCA), and human leukocyte antigen B 27 (HLA-B 27) typing were negative. Due to elevated ESR, elevated liver enzymes, and possible densification on the left lung, CT scans of the chest, abdomen, and pelvis were requested that revealed a mild thickening of the aortic wall with contrast enhancement. Similar changes were also found on the left subclavian artery and abdominal aorta. These findings were compatible with the diagnosis of GCA. Within three days, she underwent a left unilateral temporal artery biopsy under local anaesthesia, which revealed chronic granulomatous inflammation consisting of multinucleated giant cells, lymphocytes, and macrophages concentrated at the level of the internal elastic lamina as well as intimal hyperplasia, thus confirming the diagnosis of GCA. An anamnesis of the patient's symptoms revealed an episode with jaw claudication the day before hospitalization. Over the past four weeks, she had been complaining of transitory headache as well as pain in the back, neck, and shoulders. She also had bilateral prominent temporal arteries, but no tenderness had been observed on clinical examination.

### 2.2. Treatment

In the Department of Acute Psychiatry, the medical treatment initially included mood-stabilizing medication (valproate 900 mg/day and quetiapine 75 mg/day), benzodiazepines (diazepam 10 mg/day), and hypnotics (zopiclone 7.5 mg/day). She also received antibiotics (pivmecillinam) for the suspected urinary tract infection and laxatives (lactulose 20 mL) for constipation.

The diagnosis of GCA was made three weeks after readmission based on computed tomography (CT) findings and temporal artery biopsy. Pharmacological treatment was immediately prescribed. She received oral prednisolone 60 mg/day, prophylactic medication against steroid-induced gastric ulcers (pantoprazole 20 mg/day), and steroid-induced osteoporosis (calcium and vitamin D). Antiplatelet therapy, such as acetylsalicylic acid (ASA), could have been considered according to the guidelines [7], but it is unknown why it was not prescribed.

The known side-effects of steroids include mania, psychosis, and cognitive changes [8]. Accordingly, the patient had exacerbated psychotic, manic, and cognitive symptoms immediately after initiating corticosteroid treatment. She even developed illusions and visual hallucinations. Valproate was therefore gradually increased to a maximum of 1800 mg/day to control her symptoms.

The inflammatory markers gradually improved after the initiation of corticosteroids. The CRP levels declined from 66 mg/L to 37 mg/L within 48 hours and were normalized within seven days. ESR decreased to 30 mm/h during the first week of treatment and normalized within four weeks. The improvements in inflammatory markers were accompanied by clinical improvements in back pain, headache, neck pain, and stiffness.

After eight weeks of gradual dose reduction of corticosteroids, the patient's manic and psychotic symptoms rapidly improved. She had a full recovery of all psychiatric symptoms four to five months after treatment initiation.

### 2.3. Outcome and Follow-Up

Six days after steroid initiation, the patient reported unclear and blurry vision in her left eye. A finger count eye test (CF) revealed that she was able to count the examiner's fingers from a maximum distance of 30 cm as her visual field was restricted inferiorly on the temporal side. CT angiography of the cerebral arteries revealed a poorly visualized ophthalmic artery. Ischemia could not be excluded. Orbital MRI detected increased signals in the left optical nerve, compatible with optic neuritis. On retrospective analysis, the vision loss was most likely secondary to GCA. A loading dose of acetylsalicylic acid 300 mg was immediately administered, and ASA was maintained at 75 mg/day and recommended to be continued indefinitely. Intravenous methylprednisolone 500 mg/day for three days was immediately prescribed to prevent vision loss in her unaffected right eye. Oral prednisolone was paused during the use of intravenous methylprednisolone.

After discharge, patient follow-up continued for three months in order to manage her symptoms. Subsequently, she was described to have fully recovered from her psychiatric symptoms by her family. She is still, one and a half years later, followed-up by an ophthalmologist and a rheumatologist.

## 3. Discussion and Conclusions

We report a case of an atypical GCA manifestation with predominantly manic, psychotic, and cognitive features. The literature on the psychiatric effects of GCA is limited. Our patient manifested with clinical features and a clinical course that has, to our knowledge, not been described or published before. Therefore, it is possible that GCA is an underdiagnosed disease in psychiatric populations.

Our patient's physical symptoms only appeared upon closer investigation: fluctuating myalgia, jaw claudication, headache, and stiffness. Treatment with corticosteroids led to a gradual improvement of somatic symptoms. However, the symptoms of mania and psychosis initially worsened after treatment with corticosteroids, only gradually improving after 3–4 weeks of continuous treatment, indicating that the symptoms were associated with GCA.

Glucocorticoids are the backbone of GCA therapy which is aimed at suppressing immune-related GCA activity. Treatment with oral prednisolone 40 to 60 mg per day with subsequent tapering leads to remission in most patients. However, treatment with intravenous methylprednisolone should be considered for patients with acute visual loss or amaurosis fugax.

Usually, glucocorticoid treatment must be maintained for at least 2 years. Adjunctive immunosuppressive therapy, such as tocilizumab and methotrexate, should be used in selected patients who already have developed or are at a higher risk of side-effects of glucocorticoids. This includes patients with diabetes, osteoporosis, or cardiovascular diseases. Therefore, the use of adjunctive therapy should be balanced against potential treatment-related risks [7].

This atypical presentation of GCA revealed a rheumatic cause of affective, psychotic, and cognitive symptoms experienced by a patient older than 50 years. An organic cause was immediately suspected at admission; however, the final diagnosis was not determined for more than three weeks of admission.

### 3.1. Learning Points

1. GCA is the most common systemic vasculitis in people ≥ 50 years. It can present with physical and psychiatric symptoms, including mania, psychosis, cognitive dysfunctions, and visual hallucinations
2. An organic cause for cognitive, affective, and psychotic symptoms is to be considered in patients presenting with their first symptom at an age ≥ 50 years. This is especially vital if the patient presents with atypical, polymorphic, and rapidly fluctuating psychiatric symptoms
3. GCA is possibly an underdiagnosed disease in psychiatric patients. Underdiagnosed GCA can have severe and possibly fatal consequences, including blindness and stroke

## Figures and Tables

**Table 1:** Diagnostic approach and treatment of GCA according to European Alliance of Associations for Rheumatology (EULAR) recommendations [7].

Key symptoms	General symptoms (e.g., fever, weight-loss, and night sweats), jaw claudication, new-onset of persistent localized headache, acute visual symptoms (e.g., amaurosis fugax, acute visual loss), and tenderness of the superficial temporal arteries
Laboratory data	CRP, ESR, and liver enzymes
Imaging modalities	Ultrasound and MRI for temporal or other cranial arteries. Ultrasound, MRI, CT, or PET-CT for the aorta/extracranial arteries
Biopsy	Temporal artery biopsy
Pharmacological treatment	Glucocorticoids; adjunctive therapy, such as tocilizumab and methotrexate in selected patients. Antiplatelet or anticoagulant therapy should not be routinely employed unless indicated for underlying cerebrovascular and coronary heart disease and are warranted in case of vascular ischemic complications.

CRP: C-reactive protein; ESR: erythrocyte sedimentation rate; MRI: magnetic resonance imaging; CT: computed tomography; PET-CT: positron emission tomography and computed tomography.

**Table 2:** Initial laboratory test results.

Normal blood test	Normal range	Deviating blood test	Normal range
Haemoglobin 12.7 g/dL	11.7–15.3 g/dL	↑ Leucocytes 11.6 × 10^9^/L	4.1–9.8 × 10^9^/L
		↑ Thrombocytes 764 × 10^9^/L	164–370 10^9^/L
Folate 10 nmol/L	7–29 nmol/L	↑ C-reactive protein (CRP) 107 mg/L ↑ Albumin 46 g/L	0–5 mg/L 34-45 g/L
International normalized ratio (INR) 1.0	0-8–1.2	↑ Ferritin 433 *μ*g/L	20–167 *μ*g/L
Troponin-T 11 ng/L	0–14 ng/l	↑ Alkaline phosphatase (ALP) 147 U/L	35–105 U/L
NT-proBNP 152 ng/L	0–301 ng/L	↑ Alanine transaminase (ALAT) 50 U/L	10–45 U/L
Glucose, nonfasting 7.4 mmol/L	4–7.8 mmol/L	↑ Gamma-glutamyl transferase (GT) 150 U/L	10–75 U/L
Thyroid-stimulating hormone (TSH) 0.70 mIE/L	0.24–3.78 mIE/L		
Free thyroxine (FT4) 18.8 pmol/L	11.6–19.1 pmol/L		
Ethanol < 2.2 mmol/L	<2.2 mmol/L		

NT-proBNP: N-terminal pro-B-type natriuretic peptide.

## Data Availability

The datasets used and/or analysed during the current study are available from the corresponding author on reasonable request.

## Abbreviations

GCA: Giant cell arteritis
CRP: C-reactive protein
ESR: Erythrocyte sedimentation rate
MRI: Magnetic resonance imaging
CT: Computed tomography
PET-CT: Positron emission tomography and computed tomography
NT-proBNP: N-terminal pro-B-type natriuretic peptide
ANA: Antinuclear antibodies
ANCA: Antineutrophil cytoplasmic antibodies
INR: International normalized ratio
TSH: Thyroid-stimulating hormone
FT4: Free thyroxine
ALP: Alkaline phosphatase
ALAT: Alanine transaminase
GT: Gamma-glutamyl transferase.
